# Pneumonia, Arthritis, and Liver Injury: A Cardiac Diagnostic Conundrum

**DOI:** 10.7759/cureus.39505

**Published:** 2023-05-25

**Authors:** Abdul Rahman Akkawi, Ahmad Mahdi, Freidy Eid

**Affiliations:** 1 Internal Medicine, University of Kansas School of Medicine Wichita, Wichita, USA; 2 Cardiology Department, University of Kansas School of Medicine Wichita, Wichita, USA

**Keywords:** transthoracic echocardiogram(tte), transesophageal echocardiography (tee), endocarditis, shoulder septic arthritis, pulmonary valve, pulmonary valve endocarditis

## Abstract

A 40-year-old intravenous cocaine user presented with non-specific symptoms, including fever, headache, myalgias, and fatigue. After being provisionally diagnosed with rhinosinusitis and discharged on antibiotics, the patient returned with shortness of breath, dry cough, and persistent high-grade fevers. Initial workup showed multifocal pneumonia, acute liver injury, and septic arthritis. Blood cultures were positive for methicillin-sensitive staphylococcus aureus (MSSA) which led to the evaluation of endocarditis with a transthoracic echocardiogram (TTE) and transesophageal echocardiogram (TEE). TEE was performed as the initial diagnostic imaging test, and it did not show any evidence of valvular vegetation. However, given the persistence of the patient's symptoms and clinical suspicion of infective endocarditis, TTE was performed which showed a 3.2 cm vegetation on the pulmonic valve with severe insufficiency, leading to a diagnosis of pulmonic valve endocarditis. The patient was treated with antibiotics and underwent a pulmonic valve replacement surgery, which showed a large vegetation on the ventricle portion of the pulmonic valve that was replaced with an interspersed tissue valve. The patient was discharged in stable condition after improvement of symptoms and normalization of liver function enzymes. It is important to note that TTE should be considered initially as a diagnostic tool in such cases. Sometimes, a TEE may not be required if the TTE provides a sufficient assessment.

## Introduction

Infective endocarditis (IE) is characterized by microbial infection of the inner lining of the native heart valves, prosthetic valves, or implanted cardiac devices, causing inflammation and damage to the affected structures [1]. IE is a serious medical condition; if left untreated, it can lead to significant morbidity and mortality [2-3]. The aortic and mitral valves are the most commonly involved in endocarditis [4]. The common causes include bacterial infections, such as streptococcus and staphylococcus, and fungal infections [5]. Compared to left-sided IE, right-sided IE is more commonly linked to intravenous drug use (IVDU), central venous catheters, and intracardiac devices, which have become more prevalent in recent years [6]. Right-sided IE accounts for 5-10% of all cases of IE, and IVDU is considered a strong risk factor for such an infection [6-7]. It is commonly observed that the tricuspid valve is most often affected in cases of endocarditis in IVDU, followed by the mitral and aortic valves [8]. A study conducted by Mathew et al. has shown that among injection drug users, the pulmonary valve is involved in only a small number of cases [8]. IVDU increases the likelihood of introducing bacteria and other foreign organisms into the bloodstream, which can then infect the heart valves [7]. In addition, individuals who use IVDU may also have weakened immune systems, making them more susceptible to bacterial and fungal infections [7]. Endocarditis has a wide range of symptoms, which can be nonspecific and difficult to diagnose [5, 9]. Early diagnosis and treatment are essential to prevent serious complications, such as heart valve damage, heart failure, and embolic events [9]. Endocarditis symptoms can be gradual onset, and it can take weeks or even months to diagnose the condition [3]. It is important for healthcare providers to be aware of the risk factors and to consider endocarditis in individuals who present with signs and symptoms of infection, including fever, fatigue, shortness of breath, and chest pain. We herein present a case of a pulmonic valve (PV) IE presenting as multifocal pneumonia and septic arthritis with biochemical findings of acute liver injury [10].

## Case presentation

A 40-year-old male intravenous cocaine drug user presented to the hospital with fever, headache, myalgias, fatigue, shortness of breath, and a dry cough. He was initially diagnosed with rhinosinusitis and was discharged home on amoxicillin. Regardless of the antibiotic treatment, he continued to have persistent high-grade fevers, headaches, and diffuse bodily aches. The patient returned to the hospital for further evaluation, and a chest X-ray raised concerns for multifocal pneumonia. Doxycycline was added to his antibiotic regimen, and he was discharged home. A week later, the patient returned to the hospital due to a large lump on the right forearm; he was discharged with the diagnosis of thrombophlebitis and prescribed over-the-counter pain medications. 

The patient returned a week later, complaining of right shoulder swelling and pain. He was found to be septic with elevated liver enzymes. The physical examination was largely unremarkable except for a mild systolic murmur auscultated during the cardiovascular exam. MRI showed joint effusion with an enhancement of synovium. Septic arthritis diagnosis was made after shoulder fluid cultures were positive for methicillin-sensitive Staphylococcus aureus (MSSA). Additionally, blood cultures were also positive for MSSA. This prompted a transesophageal echocardiography (TEE) evaluation as the first diagnostic test, which was inconclusive.

**Figure 1:**
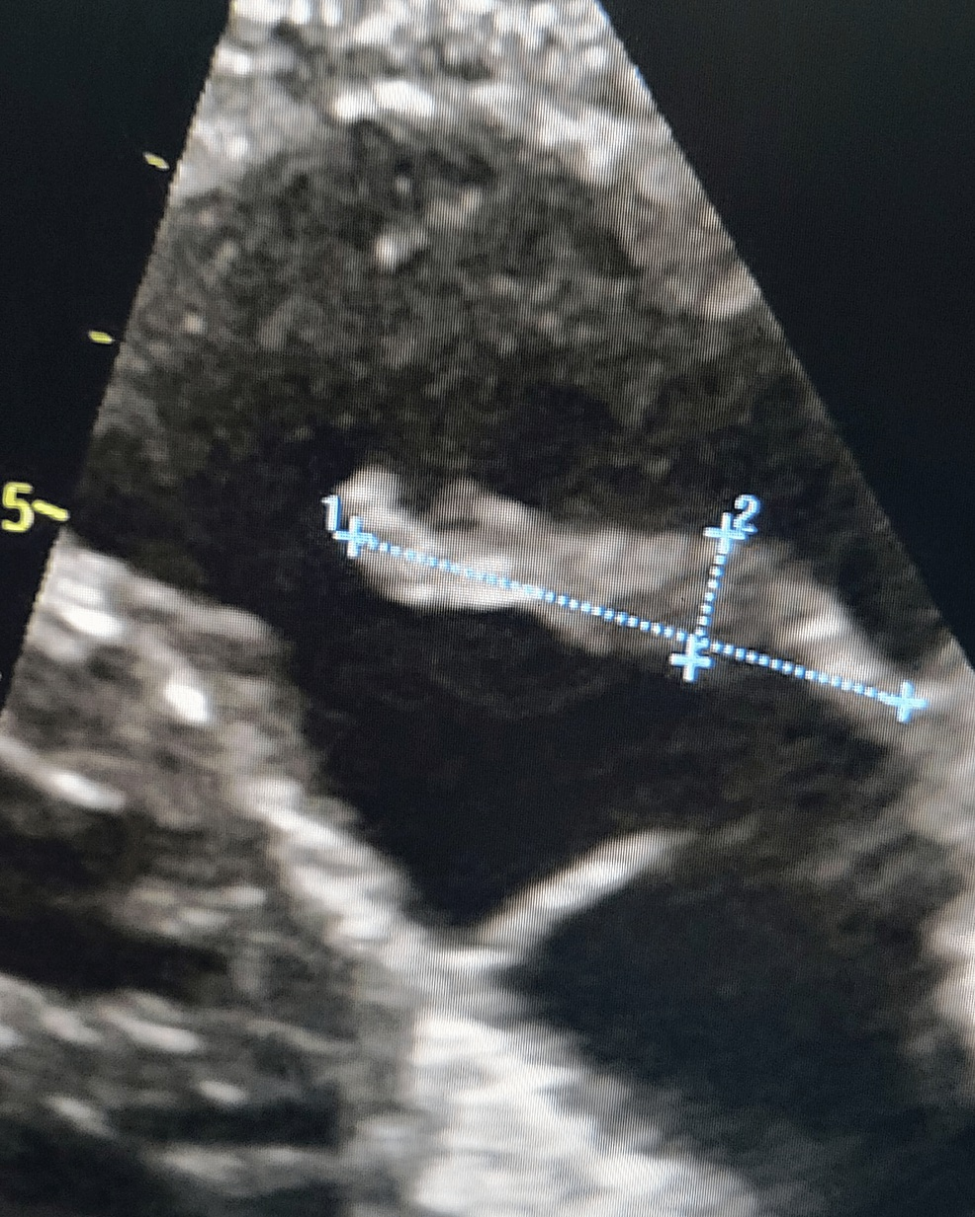
TTE showing the pulmonary valve vegetation measuring 3.2x1.1 cm TTE: transthoracic echocardiography

However, given the persistence of the patient's symptoms and clinical suspicion of infective endocarditis, transthoracic echocardiography (TTE) was performed, showing a 3.2 cm PV vegetation with severe pulmonic insufficiency (Figure 1). Consequently, the patient was started on IV nafcillin but developed a rash and was eventually switched to cefazolin.

After a week of antibiotics, the patient underwent cardiothoracic surgery for pulmonary valve replacement (PVR). Surgery showed a large vegetation on the ventricle portion of the PV, which was replaced with an interspersed tissue valve. The PV tissue was biopsied and sent for cultures, showing MSSA. After the procedure, the liver function enzyme normalized, and his condition improved significantly. The patient was discharged in stable condition on IV cefazolin for a total of six weeks.

## Discussion

PV IE is a rare but serious disease; it occurs at a higher rate in IVDU [6]. It is suggested that the initial route of infection may not be the most important factor in the development of IE, as a single contact of bacteria to heart valves is insufficient for deployment and proliferation in the endocardium [10]. Constant contact for a longer duration, such as bacteremia and sepsis, is possibly needed to cause pathological changes [10]. However, intravenous drug addicts are an exception to this trend and are more susceptible to endocarditis of the right heart valves [10]. This may be due to the several bacteria present in a single contaminated intravenous drug infusion, which can quickly deploy on the first valves encountered [9].

A study by San Román et al. showed that TEE is more sensitive than TTE in detecting pulmonic valve vegetation [11]. However, our case points out the complimentary role of TTE to TEE in diagnosing IE. While some providers may start with a TEE as a first-line, TTE could be utilized as an initial approach as it is a less invasive procedure and can complement the information obtained by a potential TEE. Therefore, some patients may not even need a TEE if the TTE provides a good enough assessment.

PV IE can lead to severe valvular insufficiency, in which the valve cannot close properly, resulting in significant blood flowing back into the right ventricle [12]. This can lead to increased pressure in the right side of the heart and the lungs, which can cause hepatic congestion and an elevation of liver enzymes, such as ALT and AST [13]. In such cases, PVR may be indicated to improve cardiac function, alleviate right heart strain, and avoid right heart failure [14]. This typically involves the surgical removal of the infected valve and the replacement with a mechanical or bioprosthetic valve. In the case we present, another indication for the surgical intervention was the size of the vegetation. The American Heart Association (AHA) recommends considering surgery for right-sided IE of a native valve when the vegetation size is larger than 10 mm to prevent embolic events [15-16].

The decision to perform PVR in patients with PV IE is complex. It requires careful consideration of various factors, including the severity of valve insufficiency, the patient's overall health, and the risk of complications associated with surgery [17]. PVR is typically reserved for patients with severe PV insufficiency experiencing significant symptoms or complications, such as heart failure, or those whose condition is not responding to medical management [17].

## Conclusions

Endocarditis is a serious medical condition that can lead to significant morbidity and mortality if not diagnosed and treated promptly. Intravenous drug use is a strong risk factor for endocarditis. This case highlights the importance of recognizing and addressing drug use as a risk factor for endocarditis in patients presenting with nonspecific symptoms. Additionally, it shows the importance of prompt diagnosis and treatment to improve outcomes for these patients and the complementary role of TTE and TEE in evaluating bacteremia. The severe PV insufficiency created by the valve changes can cause a right heart strain and may lead to right-sided heart failure. This may present with hepatic congestion and elevated liver enzymes, indicating PVR as a treatment option. The decision to perform surgery should be made in conjunction with a multidisciplinary team and the patient after carefully considering the patient's overall health and the potential risks and benefits of the procedure.
